# An evaluation of a multidisciplinary care planning tool for people with intellectual disabilities and behaviours of concern

**DOI:** 10.1177/00207640241299395

**Published:** 2024-11-29

**Authors:** Tamsin Tripp, Rebecca Goodey, Shoumitro Deb, Oliver Thomson, Jonathan Gartside, Kelvyn Hipperson, Rohit Shankar

**Affiliations:** 1 Cornwall Partnership NHS Foundation Trust, Truro, UK; 2 Imperial College London, London, UK; 3 Cornwall Intellectual Disability Equitable Research (CIDER) University of Plymouth Peninsula School of Medicine, Truro, UK

**Keywords:** Challenging behaviour, care planning, Intellectual disability, multi-agency

## Abstract

**Background:**

Multidisciplinary care planning for people with intellectual disabilities who engage in behaviours of concern (BoC) is challenging and complex. Effective collaborative understanding and action planning across all stakeholders is essential. Cornwall’s Adult Community Learning Disability Team developed a care planning tool (*Connect Behaviour*) using contemporary evidence and best practice. *Connect Behaviour* is designed to facilitate care planning by collaborative and enhanced shared understanding and is also available as an interactive web-based tool.

**Aim:**

To evaluate *Connect behaviour* using stakeholders’ experiences.

**Methods:**

Family members, care providers, advocates, social workers and professionals who had attended meetings guided by the *Connect Behaviour* care planning tool in a 3-month period were interviewed about their experiences of those meetings. This was interpreted using inductive thematic analysis.

**Results:**

Of 71 individuals identified as meeting this criterion of inclusion 61 were contactable and 27 consented to participation. These participants consisted of 17 health professionals, two parents, two social workers, three advocates, two positive behaviour support advisors within care providers, and one manager of a local care provider. The thematic analysis of participant views of *connect behaviour* generated five superordinate themes of ‘pragmatic’, ‘enabling’, ‘validation of effort’, ‘perceived lack of flexibility’ and ‘areas for development’. Further inquiry of themes highlighted subthemes of *Connect Behaviour* being ‘sensible’, ‘practical’, ‘collaborative’, ‘holistic’ and ‘action’. Conversely, some perceived a lack of flexibility in the care planning tool. Future areas for development were also identified in the data. Changes were recommended to increase the tool accessibility for people with intellectual disabilities, support stakeholders understanding of the tool, and consider utility for other clinical scenarios.

**Conclusions:**

The evaluation established general feedback to continue to use the *Connect Behaviour* as a care planning tool. The benefits of *Connect Behaviour* in comparison to other care planning frameworks needs to be understood.

## Introduction

It has been consistently recognised that people with intellectual disabilities (also called learning disabilities in the UK) face a larger number of health and behaviour-related issues than those who do not have intellectual disabilities (National Institute for Health and Care Excellence [NICE], 2018a) yet often do not receive the appropriate care planning and support they need (Gates, 2006). Successful care planning may appear to be simple, but it is an amalgamation of vast expertise and knowledge of a person, their circumstances, and effective support practices. Mansell said ‘Life for people with major disabilities supported by good services will often look quite ordinary, but this ordinariness will be the product of a great deal of careful planning and management’. (Mansell, 2007).

Between 10 and 18% of people with intellectual disabilities engage in behaviours of such a severity and frequency that they pose a critical risk to the quality of life and safety of either the person and/or those around them (Bowring et al., 2017; Deb et al., 2001; Emerson et al., 2001; Rojahn et al., 2012). Recent practice has adopted the term behaviours of concern (BoC) for this to reflect the broad range of behaviours, a preventative response to those behaviours and the context of the person in their environment (Chan et al., 2012). These behaviours can encompass physical or verbal aggression, self-injury, stereotypical behaviours, withdrawal, and destructive behaviour (Emerson, 1995; NICE, 2015 ). It usually serves a biopsychosocial function for the person, for instance conveying an unmet need such as physical and/or psychological pain, gaining control of a situation, serving a sensory purpose, or reflecting an environment that does not engage a person (Hastings et al., 2013; NICE, 2015).

This complex biopsychosocial interplay which gives rise to BoC in people with intellectual disabilities poses significant challenges to the provision of appropriate person-centred care (Barr, 2003). Factors associated with BoC can be both causal and consequential of BoC and can include biological factors such as particular genetic syndromes (Arron et al., 2011; Clarke & Deb, 2009; Folch et al., 2018; ), perceptual differences (Cooper et al., 2009; Kinnear et al., 2018), physical health conditions (Deb, 2016; De Winter et al., 2011 ; Kinnear et al., 2018), mental health concerns (Deb et al., 2021; Felce et al., 2009; Hemmings et al., 2013) and socioenvironmental factors such as trauma, isolation, loneliness, and lack of independence (Deb, 2016; Gilmore & Cuskelly, 2014; Hastings et al., 2013; Nankervis et al., 2020).

Not understanding the dynamic reasons for someone with an intellectual disability resorting to BoC may not only be detrimental to the wellbeing of the person and those around them but can increase the risk of restrictive, aversive, and excluding practices such as exclusion from community activities, loss of friends and families, physical restraint and inappropriate use of psychotropic medications (Gore et al., 2022). In extreme cases this may also lead to loss of community placement and/or hospitalisation.

It is widely recognised that people with an intellectual disability who exhibit BoC are uniquely vulnerable to overmedication. Sheehan et al. (2015) showed that around 50% of adults with intellectual disabilities are receiving psychotropic medications, often off-licence, for BoC which leads to higher instances of side effects, adverse events, and decreased quality of life (Bourgeois et al., 2010; de Kuijper et al., 2013; Matson & Mahan, 2010; Scheifes et al., 2016).

As of July 2024, over 2000 individuals with an intellectual disability and/or autism are in inpatient mental health services and, the majority engaged in behaviours that may cause risks to themselves or others (NHS Digital, 2024). Furthermore, of those with an intellectual disability and/or autism who are currently inpatients, 53% have stayed for longer than 2 years with (NHS Digital, 2024). As such, it can be said that a significant proportion of people with intellectual disabilities currently residing in inpatient services are doing so as a means of risk reduction in relation to BoC.

Indeed, there has been a substantial national focus on ensuring better care and support for people with intellectual disabilities who engage in BoC (Department of Health, 2012, 2014; NHS England, 2015b; NICE, 2015a; NICE, 2018b). There is a body of evidence to suggest that multiagency collaboration is the key to meeting people’s needs (NICE, 2015a). NHS England recommends to successfully improve the quality of life of those with intellectual disabilities, it requires multidisciplinary and multiagency working with a partnership from health teams, social care, the person with intellectual disabilities, their families, and those who care for the person. This same guideline recommends the care to be properly planned and coordinated (NHS England, 2015a).

This sophisticated level of multiagency working has the potential to have problematic dynamics within the complex system preventing the person with intellectual disability to be at the centre of the work (Atkinson et al., 2007), such as communication failures or misunderstood needs (Barr, 2003). NICE guidelines (2015a) emphasise the need to involve the person with intellectual disabilities and all other stakeholders such as families, support workers, advocates, and other professionals in the person-centred care planning (Ratti et al., 2016). It also encourages recording and sharing of the detail of the person-centred care planning among all the stakeholders including the person with intellectual disabilities and their families. Furthermore, guidelines encourage Community Learning Disability Team (CLDT) members to work in a personalised, flexible, and accessible manner while carrying out care planning (NICE, 2018b).

### The connect behaviour tool

**Figure 1.**
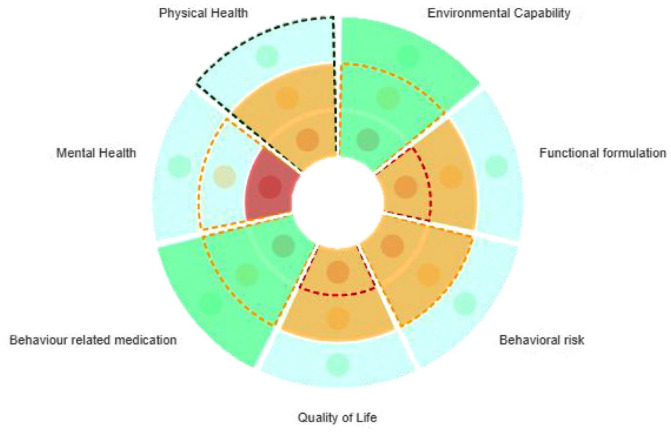
Visual representation of connect behaviour findings. Note. Solid fill represents current rating, and dashed lines the last rating of service provision in given domains

To support collaborative care planning for people with intellectual disabilities displaying BoC; Psychology, Psychiatry, and Nursing professionals within the Cornwall CLDT developed the *Connect Behaviour* tool in 2017. Based on the latest NICE guidance (NICE, 2015a, 2015b), and the Positive Behaviour Support Competence Framework (PBS Coalition, 2015 ), *Connect Behaviour* constitutes a care planning tool which enables the evaluation of service provision in line with the biopsychosocial domains that may contribute to BoC, over time. The tool enables multi-disciplinary assessment of service provision across nine key areas (see Table 1), by utilising a ‘traffic light’ system wherein the present state of support in said area is characterised as being Red (lots of work required), Amber (some further work required) or Green (very little further work required), therefore indicating the level of development required to reach optimal support in each area. It was moved to be a dynamic web-based tool supported by the NHS Trust. The technological transfer and development *of Connect Behaviour* is provided in supplementary information 1. By characterising each dimension in this way, the previous and current quality of service provision can be presented in an accessible graphical format, aiding ease of comparison (see Figure 1).

**Table 1.** Connect behaviour tool.

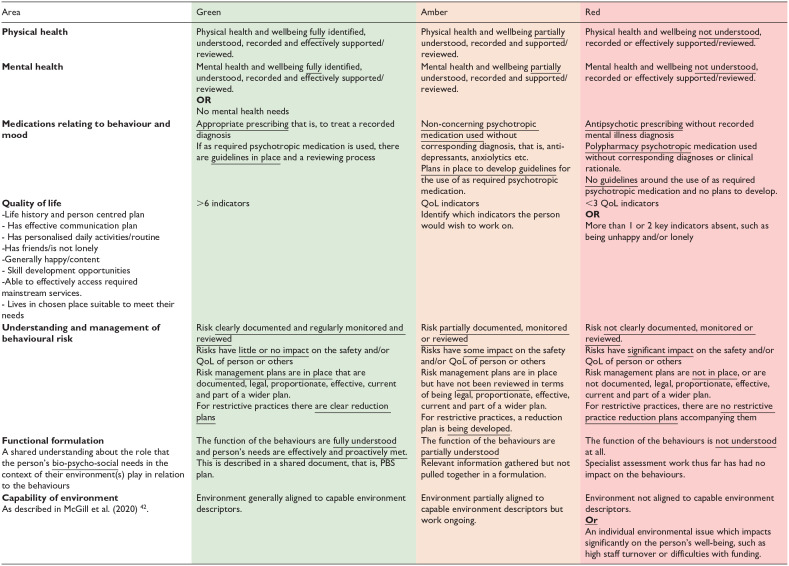

Although *Connect Behaviour* has been used locally for some time yearly there are nearly 500 webpage views., this is the first evaluation to review its use.

The present study aims to explore stakeholders’ experiences of the *Connect Behaviour* tool, through qualitative interviews undertaken with individuals who attended *Connect Behaviour* meetings for people with intellectual disabilities exhibiting BoC within Cornwall a County of the UK (population:538,000). It is believed gleaning these insights will aid in the ongoing and iterative development of the tool, with the goal of creating a framework which enables a structured, holistic review of service provision to individuals.

## Method

### Design

The present study constitutes an inductive thematic analysis of semi-structured interviews with participants as per Braun and Clarke (2006, 2021).

### Participants

To assess the impact of the tool on care planning, a purposive sample of stakeholders were interviewed who had attended at least one *Connect Behaviour* meeting that took place between 1st October and 30th December 2020. By reviewing caseload records and liaising with CLDT colleagues, a total of 71 individuals were identified as meeting this criterion of inclusion. Of these, 61 were contactable, 27 of whom consented to participation. These participants consisted of 17 health professionals in Cornwall CLDT, two parents, two social workers, three advocates, two positive behaviour support advisors within care providers, and one manager of a local care provider. During the 3-month timeframe encompassed by the present study, no individuals (i.e. patients) for whom the meetings were convened attended these meetings. No patients were involved in this study. As such, the perspectives of people with intellectual disabilities were not directly observed by the study.

### Methodological theory

In this, an exploration of participants’ experiences of using the *Connect Behaviour* tool, an aptly explorative methodology was most suitable. The constructivist underpinnings of thematic analysis (Braun & Clarke, 2006, 2021) enable the consideration of commonalities and points of difference within diverse experiences of using the *Connect Behaviour* tool, a high degree of fidelity as to explicit and implicit interview content, and the ability to explore participants’ narratives regarding *Connect Behaviour* in a naturalistic fashion which is not hindered by the methodological framework. It is widely recognised that thematic analysis yields spontaneous results, most suitable for the development of a new tool such as this (King, 2004). Thematic analysis’ keen focus on reflexivity, wherein the potential influence of the researcher on results is carefully considered, provided additional confirmation that this methodology was the most appropriate for yielding natural and valid insights into the *Connect Behaviour* tool.

### Method of data collection

Semi-structured interviews were selected due to their ability to yield rich data relevant to the topic of study, while allowing space as to not impede the interviewee in their disclosure (Agarwal, 2020). A key challenge posed by this method of data collection lays in the proper design of questions – an exercise that can feel like a trade-off between prior planning and allowing for the naturalistic flow of conversation (Potter & Hepburn, 2005). To overcome this challenge, the interviewing team collaboratively outlined a few key questions which would prompt further discussions around the key topics of interest (see Appendix 1). This approach is recognised to facilitate ‘conversations with a purpose’ (Burgess, 1984), which both investigate the topic of interest and empower interviewer and interviewee to express freely (Dicicco-Bloom & Crabtree, 2006).

### Procedure

Initially, the researchers collaboratively generated key questions to put to the participants during interviews (see Appendix 1). Once recruited, participants were interviewed one-on-one via Microsoft Teams or telephone. The researcher took notes throughout, with particular focus on collecting quotes relevant to the topics of discussion. Participants were given an unlimited amount of time to express themselves freely, yielding interviews varying between circa 10 to 30 min. All interviews conducted were included in analysis.

### Governance and ethical considerations

Approval to conduct this study was gained from the Cornwall Foundation NHS Trust audit and service evaluation team. The Cornwall Foundation NHS Trust audit and service evaluation team advised that as there was no patient-related information being sought for this project and it did not affect patient care then this was not research and therefore did not require ethical approval. This was further confirmed by the Health Research Authority decision tool which is used to discover if NHS ethical approval is required. The decision tool indicated no ethical approval was required (supplementary information 2). The study was registered with the NHS Trust as a service evaluation and validated by the Trust Clinical Quality Improvement Group.

Consent was gained from each person interviewed and it was explained that the discussion would be confidential and anonymised. After the interview participants were also asked again by the interviewer whether they would agree for parts of the discussions to be published anonymously as quotations in any peer-reviewed publication. All participants consented to this and were offered the right to withdraw at any time.

### Data analysis

Analysis followed the six stages of thematic analysis as per Braun and Clarke (2006, 2021). Due to the inductive nature of this methodology, themes emerged from the data itself. Initially, the primary researcher (TT) re-read written notes from all interviews many times as to best familiarise themselves with the data. All interview notes were subsequently coded by TT in a line-by-line fashion, thus identifying initial codes. An individual (OT) independent to the study also completed these initial stages of analysis on a random selection of interviews, and results compared as to validate the initial codes that had emerged. Furthermore, triangulation was used to establish inter-rater reliability with another author (RG); this culminated in a discussion of their independent codes, and an appraisal of them as being robustly reliable. These codes were then summarised, and categorised as by their content, and being either positive or negative in sentiment. This demonstrated how codes were interlinked and enabled overarching themes to emerge. All transcripts were subsequently re-read with the developed themes in mind to ascertain whether these themes reflected the whole data set in good faith. The themes were indeed understood to accurately reflect the positive and negative feedback that was given by participants and were displayed this in a way that each theme is distinguishable from one another. Themes were labelled in a way that best represented the essence of the data, as per the whole research team’s understanding of the data. Five key themes, each with subthemes arose from this analysis, as discussed below.

## Results

The thematic analysis identified superordinate themes of ‘pragmatic’, ‘enabling’, ‘validation of effort’, ‘perceived lack of flexibility’ and ‘areas for development’.

### -Pragmatic

The theme ‘pragmatic’ highlighted the tool adopting a logical approach. Subthemes included valuing the tool as being ‘sensible’ and ‘practical’.

#### 1a- Sensible

Participants identified that they appreciated the way in which the tool covered a broad range of relevant clinical issues in a straightforward manner. Several individuals mentioned the tool acting as an important memory aid, with the prompts ensuring certain discussions were not lost that often can be:

‘*It guides your discussion and helps you move into other areas which you might not do otherwise, sometimes can get stuck in a problem in meetings and can miss areas without Connect Behaviours*’. (P22, P1, L1-3)

‘*It prompts asking about things like communication passports and is helpful to remind of health action plan, pain profiles which is definitely helpful as these are things that could get lost in another review*’ (P20, P1, L31-33)

It was noted that not only was the tool covering a broad range of areas, but that it was also providing this thoroughly:

‘*Useful in the sense of a comprehensive overview of what’s happening*’. (P1, P1, L1)

Often professionals discussed that working with complex circumstances can be challenging and therefore valued approaches that provided ease, rather than complication:

‘*I have recommended it for at least two other people. . .really refreshing to see something used that is simple and easy but very effective*’. (P5, P1, L17-20)

*1b-Practical*

As well as the discussion around sensibility of the content of the tool, others discussed the practical nature of the structure of the tool.

The *Connect Behaviour* tool domains at the time of the study were ordered with physical health at the top of the discussion, participants tended to talk of following this order, some individuals found this beneficial to prioritise needs:

‘*It helps to give an order of things and timing of assessments, for example with challenging behaviour you may say you will do a sensory assessment but with connect behaviours you look at physical health and something could clearly be going on and that should take priority, it can help you to prioritise*’ (P25, P2, L23-26)

The tool has a discussion record to record the content and actions from the meeting, including traffic light rating. This aspect of the tool was attributed to holding a clear and formal record of information that can be reflected upon:

‘*It highlighted clearly in the traffic light where there was deficits and actions that needed to be taken*’. (P5, P1, L12-13).

‘*Connect behaviours can make sense of a very clunky system and keeps information together, it can make sure not to miss anything as you can find it easier as you can read the connect behaviour document and get a real sense of what is happening*’. (P25, P2, L5-7)

### -Enabling

The theme ‘Enabling’ incorporates the way in which the tool helps to make certain outcomes possible. This included subthemes of enabling ‘collaboration’, ‘holism’ and ‘action’.

#### 2a-Collaboration

Participants referred to the tool supporting with coordinating input from many stakeholders to work cohesively around BoC, rather than in silos:

‘*The tool is the reason it could manage 10 people in the meeting though, it allowed for more input for different disciplines, in the past there would have been several meetings, and this would then trigger referring on and having another meeting. It has cut down the amount of meetings to achieve the same thing*’ (P9, P1, L18-21).

Individuals then attributed this way of working leading to different outcomes for the person with an intellectual disability and their support systems:

‘*From the connect behaviours we looked to do a behaviour monitoring training with the team and because of connect behaviours we looked to do that jointly where previously outside of connect behaviours that would’ve been something that would have just been psychology, things were happening alongside connect behaviours but they were happening all together which is richer for the person*’ (P22, P1, L23-27)

Many also discussed collaboration being enabled by it providing a safe forum to have difficult conversations and work on relationship building:

‘*It gives you the permission to ask these things that would have been harder to weave in otherwise without being critical*’ (P20, P1, L20-21)

‘*The provider had not really had a great experience of the service before and they didn’t trust us and connect behaviours really turned things around, it helped the relationships hugely, it communicated that we are still here*’ (P21, P1, L20-22)

#### 2b-Holism

Participants mentioned the benefit of the tool facilitating discussion around a broad range of topics (7 domains) and providing a bigger picture view of BoC:

‘*Wholesome approach that it has to a person’s presentation and covers all bases and understands their needs*’. *(P11, P1, L1-2)*

The structure facilitating discussion about every aspect of a person’s presentation, quality of life or BoC with a wide range of agencies attributed some senses of being listened to:

‘*It felt like we were heard and (name of person with a learning disability) was able to get some justice*’ (P8, P1, L20).

#### 2c-Action

Participants suggested that the tool and meeting process contributed to driving action. Some suggested that this was due to the accountability that the traffic light system and recording of clear actions that are distributed to all attendees provides:

‘*Provider seemed to take on board more seriously as it holds people to account, it gives them XYZ to do clearly that is documented*’. *(*P1, P1, L15-16)

‘*It gives us clear targets and goals and these are measurable goals*’ (P6, P1, L6).

Some described the use of *Connect Behaviour* when working with particularly complex or ‘stuck’ situations to guide action when other work has not been successful:

‘*It works really well for the stuck situations with no hope and when it is so complex and multi layered*’ (P21, P1, L14-15)

Several individuals suggested examples where a need was identified and positive actions were achieved by working alongside the *Connect Behaviour* process, quite possibility due to the accountability of clear actions and planned review dates being central to the structure of the tool:

‘*An action was also SLT involvement which was not thought to be necessary before in previous meetings, the provider had lost a 10 year old SLT report which was still applicable. Without connect behaviours we would not have picked up on that need for SLT involvement*’ (P4, P1, L8-11)

‘*In connect behaviour meetings we have also identified trauma which has led to training being initiated with staff*’ (P20, P1, L11-15)

‘*For another client we had issues with the GP not doing tests we wanted but from connect behaviours she has got the covid vaccine out of it*’ (P26, P1, L12-13)

‘*I know for one person the meetings were really helpful at facilitating medication reduction, but it was also helpful in that process to have a review of things and find out they were struggling so the medication reduction could be put on pause*’ (P21, P1, L27-30)

### -Validation of effort

The theme ‘validation of effort’ encapsulates predominantly beliefs shared by participants around the traffic light rating system (green, amber, red) used to identify areas of success but also areas that require more work or understanding. This was thought to be important in terms of demonstrating positivity from professionals, with the thought in mind of motivating care providers:

‘*I also like that it is rated as it is easy in some meetings to be completely negative and focus on what has not been done, sometimes it can be hard to find but if there is a little bit of green in there it acknowledges some success and still identifies amber or red areas, it makes sure there is a balanced view so you can find positivity but also gaps*’. (P25, P1, L7-10)

People often talked of feeling as though progress or achievements were being recognised when the traffic light rating improved, as well as some security in knowing *connect behaviours* would be reviewed again in the future, with a family member stating:

‘*It gave a picture of where we were at the time, things being moved from red to amber gave a feeling of accomplishment. . .I know we will have another one as we want to move more to amber or green*’ (P9, P1, L9-35)

Others discussed the rating system as a beacon to ensure that the persons needs are the core focus that discussion is grounded in:

‘*People seemed to like the rating part of it and hopefully it takes a positive stance and keeps people at the centre*’ (P21, P1, L25-26)

### -Perceived lack of flexibility

The theme ‘perceived lack of flexibility’ describes participants opinions around the way in which the tool is delivered. The tool was intended for use in a flexible manner however many participants described feeling driven by the tool or prescribing it in a rigid manner, impacting on flow of spontaneous discussion:

‘*You feel like you need to pull it back to the structure which makes it less fluid*’ (P20, P1, L7).

Several participants wondered about the order in which domains are discussed and prioritised despite this order being open to interpretation, this appeared to be impacted by what multidisciplinary profession that the participant was aligned most with:

‘*I do wonder about the order of it as I mainly find the sections after physical health and medication which is at the start to be really meaningful*’ (P12, P1, L1-3).

‘*I sometimes wonder whether the order could be moved around a bit, sometimes that order is good with physical health at the start, it does seem to flow in that order. . .I have wondered before whether capability of environment should be at the start as without a capable environment all the other domains are never going to work*’ (P25, P2, L12-17)

Elsewhere in the analysis some participants described benefitting from the thorough nature of the tool (Pragmatic), however conversely some commented on the length of time in which this can take:

‘*It can also be quite long as there is lots to go through, sometimes maybe it needs to be two meetings*’ (P20, P1, L36-37)

Again, that contrast in benefit of use of time was further contemplated:

‘*Never before used 3 meetings for one connect behaviour but it does spark the emotions that are sometimes really needed*’ (P21, P2, L2-4)

### -Areas for development

Overall, all 27 participants said they would use the tool again either as it is already used or with some adjustments however several participants referred to ‘areas for development’, with the majority of these ideas fuelled by participants not understanding the tool or its processes fully.

Several participants showed awareness of there being an easy read version of the tool but still felt that inclusion of people with intellectual disabilities in the *Connect Behaviour* process was unfortunately limited or could be further aimed towards:

‘*In an ideal world we could meaningfully include the person, I don’t know how realistic that would be though*’. *(P19, P1, L14-15)*

‘*At the start the client did not want to come to the meetings so (name of professional) met with them and went through the domains and then took this back to the meetings and read out what she said, the client said that they wanted support workers to listen to me, this had quite a big impact . . .perhaps there should be a note at the beginning asking whether the client has been consulted*’ *(P21, P2, L10-19)*

Some also felt that current versions of the tool were difficult to understand for families, demonstrating the accessibility of the tool requiring further development or explanation:

‘*It took a while to understand it, even the simplified one was hard. . .it is alright for the psychologists and psychiatrists but what I think is imagine when I die and it will be my son who will take over this role and he doesn’t know about all this, so it needs to be simplified more*’ (P8, P1, L2-8)

Interestingly, demonstrating some widespread misunderstanding of the tool, several professionals also expressed a desire for there to be further clarity, description and training to support understanding or minimise some of the ‘perceived lack of flexibility’:

‘*I feel that consistency is missing in when connect behaviour meetings are used and when does it come in*’ (P23, P1, L9-10)

‘*For many colleagues there is a lack of clarity in what it actually is, lots of situations may benefit from connect behaviours thinking but if it is not set up in the right way it can limit its helpfulness*’ (P22, P1, L10-11)

Finally, the current version is utilised for supporting BoC, however individuals considered whether the tool would be applicable or adapted to be used in other scenarios:

‘*Connect behaviour may not fit with crisis, it could be tweaked to fit a crisis and be quicker*’ (P12, P1, L17-18).

‘*I think there is scope for widening it out to stuck situations or mental health, it can be used wider than behaviours that challenge*’ (P21, P1, L42-43)

## Discussion

This evaluation aimed to understand the experiences of professionals, family members and care providers using a care planning tool to support BoC used and developed in the Adult CLDT in Cornwall. Five themes and five subthemes emerged from the analysis of this interview data. This included superordinate themes of the tool being ‘*pragmatic*’, ‘*enabling*’ and providing ‘*validation of effort*’, however that there is also ‘*perceived lack of flexibility*’ and ‘*areas for development*’.

The benefits of the *Connect Behaviour* tool identified in the data parallel to literature for use of care planning processes in other clinical settings including children’s, mental health and dementia however literature regarding this in intellectual disabilities is more sparce (Doody et al., 2019). Interestingly there is qualitative evidence to suggest that there is a view that within mental health, meetings without clear planning, organisation or chairing are often poorly managed but that organisation and clear agendas (which *Connect Behaviour* has been described to possess in the data) can support quality of decision making (Borrill et al., 2000). In work with children, it has been identified that structured care planning can incite working together, holistic consideration and sharing common goals (Carter et al., 2007). Many participants of the current study valued the formality and recording of the review process for supporting accountability of action taking which is also attributed in children’s work as factors that promote successful multi agency working (Sloper, 2004). There have also been recommendations within dementia care planning around understanding the whole person and what is important to them and ensuring that this is recorded (Piers et al., 2018) which some participants stated that the *Connect Behaviour* process can facilitate for people with intellectual disabilities.

Regarding the validation of effort that was attributed to the *Connect Behaviour* process, it is familiar across intellectual disability services and wider healthcare to use a RAG (Red, Amber, Green) rating system (Mottershead & Woodrow, 2019). Respondents generally commented on this being used positively, rather than a way in which to criticise. It is recommended to work across varying organisations and have a strong focus on outcomes within care pathways for individuals (NHS Commissioning Board, 2013). Participants also reflected on the way in which progress on the rating system promoted a feeling of achievement, continuous evaluation of effectiveness of interventions for people with intellectual disabilities is what is recommended on an ongoing basis (Denne et al., 2020).

Conversely, the importance of recognising the constructive themes in this data is imperative. This is highlighted by the evidence that within mental health teams, if professionals experience team processes as positive, these processes are viewed as more positive by other stakeholders and more innovative and effective within teams (Borrill et al., 2000).

Participants that viewed the tool as having ‘perceived lack of flexibility’ and having ‘areas for development’ referred to the wish to meaningfully include people with intellectual disabilities in the *Connect Behaviour* process more frequently. Evidence broadly suggests that it is valuable to involve individuals in their own care planning but that this is done sporadically and inconsistently in healthcare (Anthony & Crawford, 2000). The Valuing People report stated that involving people with intellectual disabilities in their care planning is facilitative of increasing their sense of power and control (Department of Health, 2001). NHS England further strengthened that position in the 2019 Long Term plan where they stated that NHS services need to strive to include people with a intellectual disability in the experience of outcomes from their care and treatment (NHS England, 2019).

Service user involvement is known to be increased by an improvement in accessibility of documentation used (Anthony & Crawford, 2000) which is an aim portrayed following this evaluation. People with intellectual disabilities have also attributed the importance of others to have the opportunity to advocate for them as well, particularly in the context of meetings (Hoole & Morgan, 2011). As for this study also not having any participants with intellectual disabilities due to not being present in the participant pool, it has been suggested that a barrier to service user involvement is the lack of information on its outcomes (Kent & Read, 1998). This could be a further area of research.

## Limitations

It is unclear whether the benefits identified in this qualitative evaluation are exclusive to the *Connect Behaviour* process as this may be more so the consequence of multiagency care planning in general and there has been no comparison to other known care planning tools in this evaluation. Evidence suggests that higher levels of attendance at meetings associates to quality of patient care however this could be said of any meeting forum (Borrill et al., 2000). Some state that there should be more focus on real changes in people’s lives rather than a focus on care planning as planning alone may not achieve outcomes (Mansell & Beadle-Brown, 2004). This evaluation was an initial exploration and therefore outcome measures on quality of life, prevalence of behaviours of concern and incidences of restrictive practice or medication use was not explicitly measured.

The volunteer sampling method and short 3-month period of inclusion utilised for this evaluation likely flawed opportunities for seeking more varied or constructive perspectives needed to hold more certainty on effectiveness of the tool. Similarly, whilst some participants may have been reflecting on use of the tool for a longer time period, many had shorter term experience of *Connect Behaviour* so the results cannot be generalised to longer term use of the tool which is often required for understanding behaviours of concern. A further limitation includes that the evaluation was not completed independently of the CLDT and therefore may have been subject to bias of participants being conscious of the way in which their feedback may impact on relationships with the team despite being assured of this.

Finally, this evaluation excluded direct participation from people with intellectual disabilities and the number of parents/carers that is, patient stakeholders too were relatively disproportionate to the number of professionals participating. This is because the design of the evaluation was focussed on exploring *connect behaviours* working particularly in helping CLDT formulate and care plan complex situations. It is however important that further development of the tool be inclusive of people with intellectual disabilities and their carers to examine how *connect behaviours* can engage and empower meaningful communication and problem synthesis.

## Implications for practice

### Implications for clinical practice

Due to the results discussed and all 27 participants of this evaluation having willingness to continuing to use the *Connect Behaviour* tool in its current form or with minor adjustments, it is endeavoured to ensure the tool is reflective of more recent literature, as well as being more accessible and understandable to use.

Regarding the wish to more meaningfully include people with intellectual disabilities in the *Connect Behaviour* process and ensure it is understandable to all stakeholders, there has been further adaptation to the easy read version of the tool following this evaluation including explicitly encouraging involvement from advocates. Trialling going through this version with those with intellectual disabilities who engage in BoC has started to occur, inciting some interesting insights which should be further investigated.

Several participants reflected on the wish to further understand how to use the tool or some misunderstanding on using it flexibly to meet clinical needs. Further training sessions to the CLDT and adaptations to the *Connect Behaviour* descriptions have been made but further consideration should be made as to whether a manual on when it could be used, how it is used, and scoring would be beneficial. A video demonstrating it’s use would be optimal.

The tool could be further adapted and evaluated in its effectiveness with other situations including crisis, mental health and transition planning. This may also address some of the data around requiring versions to be used in a quicker manner at times however caution should be held around ensuring richness is not lost when doing so.

Consideration of updating *Connect Behaviour* based on more recent literature should be an ongoing process which has been started by incorporating evidence around the capable environment values (McGill et al., 2014, 2020) recommended by the NHS, NICE and the Royal College of Psychiatrists (NHS England, 2015a; NICE, 2015a; Royal College of Psychiatrists, 2016).

### The digital interface

There has also been a significant shift in the rigour of the regulatory environment for digital health technologies. Accessibility requirements for public sector bodies is the most well established having been in force since 2018. Importantly, in addition to meeting the technical requirements of the regulations such as WCAG2.2. The Government Service Manual provides helpful guides which include fully integrating service users in the design process and design principles covering a range of accessibility needs. Furthermore, the Digital Technology Assessment Criteria (DTAC) combines previously disparate safety processes into a single approach. The challenge for future development of the tool will be to combine the best of the literature, rigorous digital approvals and the need to engage and involve users to ensure that the tool can meet and respond to their diverse needs.

### Implications for research

Research needs to determine whether *Connect Behaviour* can influence better outcomes for people with intellectual disabilities and challenging behaviours, influence reduced psychotropic prescribing prescribed for nonpsychiatric reasons, be regionally scaled and implemented in local secondary care services and be used by families and carers to help identify causes of BoC. This can be achieved by iterative testing and formative evaluation. People with intellectual disabilities themselves were not directly part of the evaluation and thus NHS ethics was not required. However, it is imperative that future studies include people with intellectual disabilities and would be required to satisfy research regulations as needed.

## Conclusion

Clinicians working with people with intellectual disabilities will have a systematic evidence-based tool that supports delivering person-centred care. *Connect Behaviour* identified nine dimensions of response that are clinically and holistically important in the understanding of BoC in people with intellectual disabilities. It aims to redirect attention from reflex actions such as medicating to a person-centred formulation and plan. It provides a prototype to outline theoretical process measures and evidence-based outcome frameworks visually. The benefits for people with intellectual disabilities, their families and carers include reducing or preventing the prescribing of unnecessary psychotropic medications, improved quality of life, morbidity and mortality outcomes.

## Supplemental Material

sj-docx-1-isp-10.1177_00207640241299395 – Supplemental material for An evaluation of a multidisciplinary care planning tool for people with intellectual disabilities and behaviours of concern Supplemental material, sj-docx-1-isp-10.1177_00207640241299395 for An evaluation of a multidisciplinary care planning tool for people with intellectual disabilities and behaviours of concern by Tamsin Tripp, Rebecca Goodey, Shoumitro Deb, Oliver Thomson, Jonathan Gartside, Kelvyn Hipperson and Rohit Shankar in International Journal of Social Psychiatry

sj-docx-2-isp-10.1177_00207640241299395 – Supplemental material for An evaluation of a multidisciplinary care planning tool for people with intellectual disabilities and behaviours of concern Supplemental material, sj-docx-2-isp-10.1177_00207640241299395 for An evaluation of a multidisciplinary care planning tool for people with intellectual disabilities and behaviours of concern by Tamsin Tripp, Rebecca Goodey, Shoumitro Deb, Oliver Thomson, Jonathan Gartside, Kelvyn Hipperson and Rohit Shankar in International Journal of Social Psychiatry

## Appendix 1

Semi Structured interview schedule

1. What was good about the meeting using the *Connect Behaviours* tool?
2. What was not so good about the meeting using the *Connect Behaviours* tool?
3. How well did *Connect Behaviours* meetings facilitate positive action for the person, please give some examples of this?
4. Do you think the same actions would have been achieved without using the *Connect Behaviours* tool in the meeting? Please expand.
5. What do you think the *Connect Behaviours* meeting helped to achieve for the person or those supporting the person that would not have happened ordinarily in a review meeting?
6. Is there anything you would change about the *Connect Behaviours* tool or anything you would do differently in *Connect Behaviours* meetings?
7. Is the *Connect Behaviours* framework something you would want to be used again. If so why? If not why not?

**Appendix 2.** Superordinate and subordinate themes and corresponding quotes.

Themes and Subthemes	Example quotations
**1. Pragmatic**	
a. Sensible	• *It guides your discussion and helps you move into other areas which you might not do otherwise, sometimes can get stuck in a problem in meetings and can miss areas without Connect Behaviours (P22, P1, L1-3)* • *It prompts asking about things like communication passports and is helpful to remind of health action plan, pain profiles which is definitely helpful as these are things that could get lost in another review (P20, P1, L31-33)* • *Useful in the sense of a comprehensive overview of what’s happening. (P1, P1, L1)* • *I have recommended it for at least two other people. . .really refreshing to see something used* that is *simple and easy but very effective. (P5, P1, L17-20)*
b. Practical	• *It helps to give an order of things and timing of assessments*, for example *with challenging behaviour you may say you will do a sensory assessment but with connect behaviours you look at physical health and something could clearly be going on and that should take priority, it can help you to prioritise (P25, P2, L23-26)* • *It highlighted clearly in traffic light where there was deficits and actions that needed to be taken. (P5, P1, L12-13).* • *Connect behaviours can make sense of a very clunky system and keeps information together, it can make sure not to miss anything as you can find it easier as you can read the connect behaviour document and get a real sense of what is happening. (P25, P2, L5-7)*
**2. Enabling**	
a. Collaboration	• *The tool is the reason it could manage 10 people in the meeting though, it allowed for more input for different disciplines, in the past there would have been several meetings, and this would then trigger referring on and having another meeting. It has cut down the amount of meetings to achieve the same thing (P9, P1, L18-21).* • *From the connect behaviours we looked to do a behaviour monitoring training with the team and because of connect behaviours we looked to do that jointly where previously outside of connect behaviours that would’ve been something that would have just been psychology, things were happening alongside connect behaviours but they were happening all together which is richer for the person (P22, P1, L23-27)* • *It gives you the permission to ask these things that would have been harder to weave in otherwise without being critical (P20, P1, L20-21)* • *The provider had not really had a great experience of the service before and they didn’t trust us and connect behaviours really turned things around, it helped the relationships hugely, it communicated that we are still here (P21, P1, L20-22)*
b. Holism	• *Wholesome approach that it has to a person’s presentation and covers all bases and understands their needs. (P11, P1, L1-2)* • *It felt like we were heard and (name of PWLD) was able to get some justice (P8, P1, L20).*
c. Action	• *Provider seemed to take on board more seriously as it holds people to account, it gives them XYZ to do clearly* that is *documented.(P1, P1, L15-16)* • *It gives us clear targets and goals and these are measurable goals (P6, P1, L6).* • *It works really well for the stuck situations with no hope and when it is so complex and multi layered (P21, P1, L14-15)* • *An action was also SLT involvement which was not thought to be necessary before in previous meetings, the provider had lost a 10* *year old SLT report which was still applicable. Without connect behaviours we would not have picked up on that need for SLT involvement(P4, P1, L8-11)* • *In connect behaviour meetings we have also identified trauma which has led to training being initiated with staff (P20, P1, L11-15)* • *For another client we had issues with the GP not doing tests we wanted but from connect behaviours she has got the covid vaccine out of it (P26, P1, L12-13)* • *I know for one person the meetings were really helpful at facilitating medication reduction, but it was also helpful in that process to have a review of things and find out they were struggling so the medication reduction could be put on pause (P21, P1, L27-30)*
**3. Validation of effort**	• *I also like that it is rated as it is easy in some meetings to be completely negative and focus on what has not been done, sometimes it can be hard to find but if there is a little bit of green in there it acknowledges some success and still identifies amber or red areas, it makes sure there is a balanced view so you can find positivity but also gaps. (P25, P1, L7-10)* • *It gave a picture of where we were at the time, things being moved from red to amber gave a feeling of accomplishment. . .I know we will have another one as we want to move more to amber or green (P9, P1, L9-35)* • *People seemed to like the rating part of it and hopefully it takes a positive stance and keeps people at the centre (P21, P1, L25-26)*
**4. Perceived lack of flexibility**	• *You feel like you need to pull it back to the structure which makes it less fluid (P20, P1, L7).* • *I do wonder about the order of it as I mainly find the sections after physical health and medication which is at the start to be really meaningful (P12, P1, L1-3).* • *I sometimes wonder whether the order could be moved around a bit, sometimes that order is good with physical health at the start, it does seem to flow in that order. . .I have wondered before whether capability of environment should be at the start as without a capable environment all the other domains are never going to work (P25, P2, L12-17)* • *It can also be quite long as there is lots to go through, sometimes maybe it needs to be two meetings (P20, P1, L36-37)* • *Never before used 3 meetings for one connect behaviour but it does spark the emotions that are sometimes really needed (P21, P2, L2-4)*
**5. Areas for development**	• *In an ideal world we could meaningfully include the person, I don’t know how realistic that would be though. (P19, P1,L14-15)* • *At the start the client did not want to come to the meetings so (name of professional) met with them and went through the domains and then took this back to the meetings and read out what she said, the client said that they wanted support workers to listen to me, this had quite a big impact . . .perhaps there should be a note at the beginning asking whether the client has been consulted (P21, P2, L10-19)* • *It took a while to understand it, even the simplified one was hard. . .it is alright for the psychologists and psychiatrists but what I think is imagine when I die and it will be my son who will take over this role and he doesn’t know about all this, so it needs to be simplified more (P8, P1, L2-8)* • *I feel that consistency is missing in when connect behaviour meetings are used and when does it come in (P23, P1, L9-10)* • *For many colleagues there is a lack of clarity in what it actually is, lots of situations may benefit from connect behaviours thinking but if it is not set up in the right way it can limit its helpfulness (P22, P1, L10-11)* • *Connect behaviours may not fit with crisis, it could be tweaked to fit a crisis and be quicker (P12, P1, L17-18).* • *I think there is scope for widening it out to stuck situations or mental health, it can be used wider than behaviours that challenge (P21, P1, L42-43)*
