# Supplementary material for: An evaluation of a multidisciplinary care planning tool for people with intellectual disabilities and behaviours of concern
Source: Int J Soc Psychiatry. 2024 Nov 29;71(4):655–69. doi: 10.1177/00207640241299395 (PMC12171078; doi:10.1177/00207640241299395)

# Connect Behaviours Webpage Technical Overview

## Background

The web page for connect behaviours was developed by the Cornwall Partnership NHS Foundation Trust (CPFT) SQL team in 2019. The goal was to create a minimum viable product that would accompany overall multidisciplinary care planning for people with intellectual disabilities and behaviours of concern. The clinical development was led by Professor Rohit Shankar and Dr Rebecca Goodey.

## Design

The requirement was for something simple that could be used both inside and outside of the Trust, which would serve as a visual aid to the care planning tool proposed. After much discussion between the CFT SQL team, the Adult Learning Disability Service, and Cornwall IT Services (CITS) over what was needed of the program, timeframes and capacity, and practicality, it was decided that a simple web page utilising HTML, JavaScript and Cascading Style Sheets  (CSS) should be used, that could be deployed as a stand-alone web page from a CITS managed web server.

For data protection/security reasons it was decided that the web page should not store any information or provide functionality for entering free text, that there should not be a “back end” database accessible by the program, and that third party code libraries should not be used. This necessitated the need for the following considerations:

- The program would be limited in overall functionality
- User entry fields would be limited to two date fields
- Data would not be stored via the web page itself
- The user of the program would need to save/print the completed session rather than save on a server hosted by the Trust/CITS
- As a minimum, the program would need to display the rag rating for a current assessment, and previous assessment, to articulate the change in rag rating for specific groupings across both assessments
- The “Traffic Light” criteria of the care planning tool – used as a point of reference (via tool tip) by the user of the program - would need to be hard coded into the underlying code itself, rather than pulled from a database

## How to Use

1. Navigate to the following web page
2. Click on the box in the top left of the page with the text “Previous Assessment Date: Click Here to Enter” (see figure 1 below)
3. A Dialogue box will open in the top middle of the page allowing the user to enter a date. If a format other than DD/MM/YYYY is entered, the dialogue box will request the user enters a valid date in that format
4. Follow the same steps for the box with the text “Current Assessment Date: Click Here to Enter” (found below the Previous Assessment Date Box)
5. To update the rag rating on the visual itself for the current or previous assessment, select the “Click Here” square next to the relevant assessment date. The box will turn green (see fig.2)
6. Click on the respective colour coded buttons in the central visualisation to populate it. Hovering the cursor over the respective button will display a text box detailing the criteria for that specific area and rag rating. Previous assessments will show a dotted line, current assessments will show a straight line (fig.3).
7. Once the visualisation has been populated for both assessments, click on the box with the text “Click Here For Print View” to render the visualisation in a size that can be printed as an A4 pdf. Once the print preview has been selected, neither the visualisation or assessment dates can be updated (fig.4).

Fig.1:


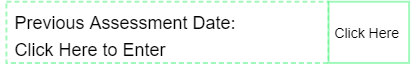


Fig.2


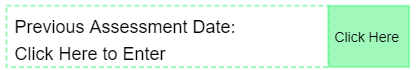


Fig.3


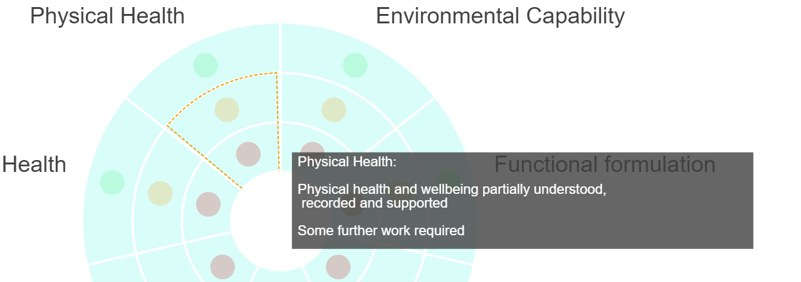


Fig.4


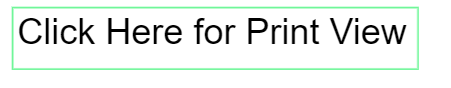

Supplement: sj-docx-1-isp-10.1177_00207640241299395 – Supplemental material for An evaluation of a multidisciplinary care planning tool for people with intellectual disabilities and behaviours of concern [file sj-docx-1-isp-10.1177_00207640241299395.docx]
