# Supplementary figures and images for: An evaluation of a multidisciplinary care planning tool for people with intellectual disabilities and behaviours of concern
Source: Int J Soc Psychiatry. 2024 Nov 29;71(4):655–69. doi: 10.1177/00207640241299395 (PMC12171078; doi:10.1177/00207640241299395)

**Supplementary information 2**


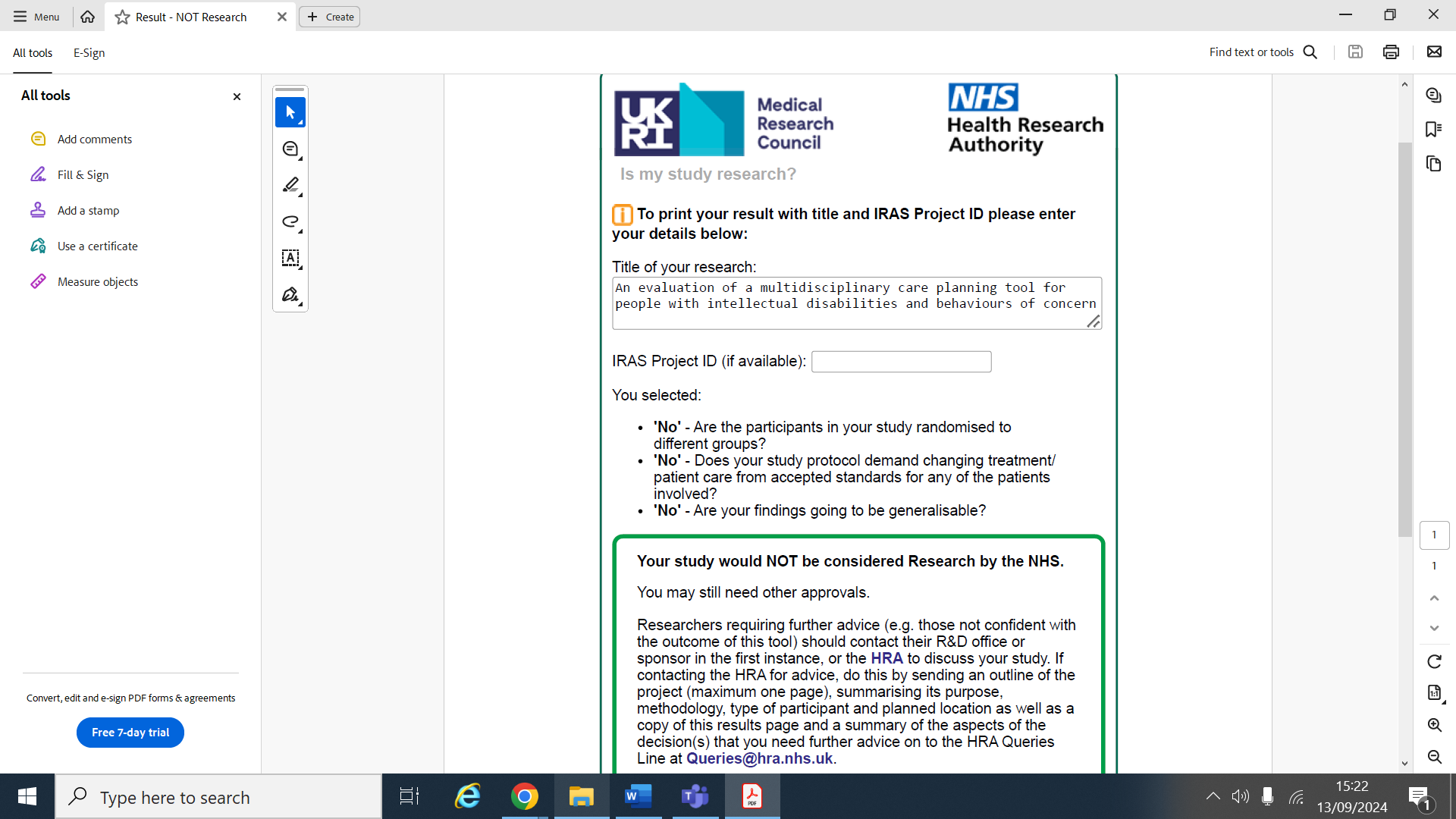

Supplement: sj-docx-2-isp-10.1177_00207640241299395 – Supplemental material for An evaluation of a multidisciplinary care planning tool for people with intellectual disabilities and behaviours of concern [file sj-docx-2-isp-10.1177_00207640241299395.docx]
